# Reuse of Textile Waste in the Production of Sound Absorption Boards

**DOI:** 10.3390/ma16051987

**Published:** 2023-02-28

**Authors:** Sigitas Vėjelis, Saulius Vaitkus, Arūnas Kremensas, Agnė Kairytė, Jurga Šeputytė-Jucikė

**Affiliations:** Building Materials Institute, Faculty of Civil Engineering, Vilnius Gediminas Technical University, Linkmenų Str. 28, LT-08217 Vilnius, Lithuania

**Keywords:** woollen yarn production waste, waste reuse, acoustical characteristics, sound absorption, noise reduction coefficient

## Abstract

Textile waste is formed in various stages, from the preparation of raw materials to the utilisation of textile products. One of the sources of textile waste is the production of woollen yarns. During the production of woollen yarns, waste is generated during the mixing, carding, roving, and spinning processes. This waste is disposed of in landfills or cogeneration plants. However, there are many examples of textile waste being recycled and new products being produced. This work deals with acoustic boards made from waste from the production of woollen yarns. This waste was generated in various yarn production processes up to the spinning stage. Due to the parameters, this waste was not suitable for further use in the production of yarns. During the work, the composition of waste from the production of woollen yarns was examined–namely, the amount of fibrous and nonfibrous materials, the composition of impurities, and the parameters of the fibres themselves. It was determined that about 74% of the waste is suitable for the production of acoustic boards. Four series of boards with different densities and different thicknesses were made with waste from the production of woollen yarns. The boards were made in a nonwoven line using carding technology to obtain semi-finished products from the individual layers of combed fibres and thermal treatment of the prepared semi-finished product. The sound absorption coefficients in the sound frequency range between 125 and 2000 Hz were determined for the manufactured boards, and the sound reduction coefficients were calculated. It was found that the acoustic characteristics of soft boards made from woollen yarn waste are very similar to those of classic boards or sound insulation products made from renewable resources. At a board density of 40 kg/m^3^, the value of the sound absorption coefficient varied from 0.4 to 0.9, and the noise reduction coefficient reached 0.65.

## 1. Introduction

About 150 million tons of textile waste are generated worldwide each year [1]. Each year, about 9.35 million tons of textile waste is collected in the European Union and more than 17 million tons in the United States each year [2,3]. In theory, approximately 95% of textile waste can be recycled, but in practice the recycling rate is very low [4]. According to American scientists, 66% of the collected waste is sent to landfills, 19% is combusted with energy recovery, and only 15% is recycled [5]. According to Li et al. [4], about 10–15% of textile waste is recycled in China and about 25% in the European Union. Researchers point out that some of the textile waste from individual consumers remains uncollected and that efforts are being made to make the collection of textile waste more efficient by 2025 [6]. Different fibre components require different reuse methods later. To realize targeted recycling, the composition and content of waste textiles must be known [7,8]. Currently, the sorting of textile waste is mainly based on manual operation. Manual sorting of textile waste based on the fibre material content listed on the product labels is slow and often unreliable because the labels may have been removed, worn out, or have incorrect information. The development of automatic sorting systems has been intensive in the last decade [9,10].

Several groups of scientists evaluated automatic sorting technologies and identified their limitations [11,12]. The authors identified properties of fabrics that led to non-matching recognition during automatic sorting. In most cases, it was the result of fabrics being coated with various chemical coatings and multi-layered fabrics. Furthermore, it was stated that ageing causes such chemical changes that hampered the recognition of the type of fibres.

In the world of material recycling, 100% pure materials have a higher value than blends. For this reason, the sorted groups include categories such as 100% cotton, 100% wool, 100% polyester, and 100% acrylic. After these categories, the sorters identify the most commonly used blends, such as cotton-polyester, polyester-wool, and wool-acrylic. After processing, mixed and unsuitable fibres for the textile industry are used in other industries. In reference [13], the authors describe a wide range of uses of various waste materials for construction materials. A lot of attention has been paid to studying various composites with textile waste. Several authors investigated the use of textile fibres as a reinforcement in cementitious composites [14,15,16]. Test results showed excellent results in terms of increasing load capacity and reducing cracks. Natural fibres obtained from textile waste are mainly used in the manufacture of thermal insulation or acoustic mats or boards [17].

In a research paper [18], scientists reported the development of a sound-absorbing material from a mixture of cotton and polyester waste. Natural rubber was used as a binder. The compression moulding technique allowed the rubber to melt and bind to the textile waste. The researchers formed the products using different compression levels, temperatures, and specimen thicknesses. Studies of the specimens obtained showed that the obtained products had very good sound absorption characteristics. The sound absorption coefficient in the high-frequency range was 0.7–0.9, and the noise reduction coefficient varied in the range of 0.5–0.7.

Patnaik [19] reported a study on thermal and sound insulation specimens developed from waste wool and recycled polyester fibres for building industry applications. The density of the specimens ranged from 58.8 to 66.7 kg/m^3^. All specimens were made using needle-punching technology. All developed specimens showed good sound absorption properties across the entire frequency range of 50–5700 Hz. Sound absorption was lower at low frequencies of 50–1000 Hz and increased from the medium frequency range of 1000–2000 Hz to the high-frequency range of 200–5700 Hz for all specimens.

In a review study [20], the researchers analysed the use of various wastes for the development of composites with good thermal and acoustic properties. The authors analysed high- and low-density composites. In this overview, the authors concluded that many waste products from various categories and production sources can be used as thermal and sound insulation materials.

An analysis has been made [21] of the environmental impact of traditional insulation materials and materials made from renewable raw materials during the production, installation, and dismantling stages. In the study, they provide a comprehensive assessment of material flows, environmental impacts, and circular economy strategies. The authors noted that lacking recycling systems and incentives for recycling, current installation and deconstruction practices, as well as the legally required incineration of oil-based insulation materials, all contribute to low material circularity. The authors stated that the three aspects of contaminants, end-of-life, and type of materials are crucial in designing a more sustainable and circular system.

The use of textile waste in the production of construction materials makes both environmental and economic indicators more meaningful. In particular, fibres used in the textile industry are treated with more environmentally friendly flame retardants [22,23], whereas environmentally harmful additives are still used in the construction industry [24,25]. In addition, the use of textile waste for construction no longer requires an additional charge for additives and a series of coating operations, and the preparation of the waste-derived materials themselves is significantly cheaper than the preparation of new raw materials.

A novel contribution of the work is the evaluation of the suitability of woollen yarn production waste for the production of acoustic boards. It includes the determination of board production parameters and evaluation of the acoustic characteristics of the created products. During this work, the quality and parameters of the waste generated during the production of woollen yarns were evaluated. Soft fibreboards based on textile fibres were created using polylactide as a binding material, and the relationship between acoustic characteristics by evaluating the densities and thicknesses of the obtained specimens were studied.

## 2. Materials and Methods

### 2.1. Raw Materials

Woollen yarn production waste was obtained from the textile company UAB Danspin, Lithuania. This waste was generated during the mixing, carding, roving, and spinning processes. During the yarn production process, raw wool was coated with insecticide to control moths. The waste from the production of woollen yarn prior to the production of boards was not coated with insecticide. Polylactide was used as the binding material. Polylactide was obtained from NatureWorks LLC (Minnetonka, USA). The average length of the polylactide was 51 mm and the average diameter was 27 µm.

### 2.2. Determination of Woollen Yarn Waste Composition

The composition of the woollen yarn waste was determined according to ISO 1833-1, ISO 1833-4, ISO 1833-7, and ISO 1833-11 [26,27,28,29] standards.

### 2.3. Production of Soft Boards

Soft acoustic boards were produced in the non-woven production line of the textile company UAB Danspin, Lithuania.

**Figure 1:**
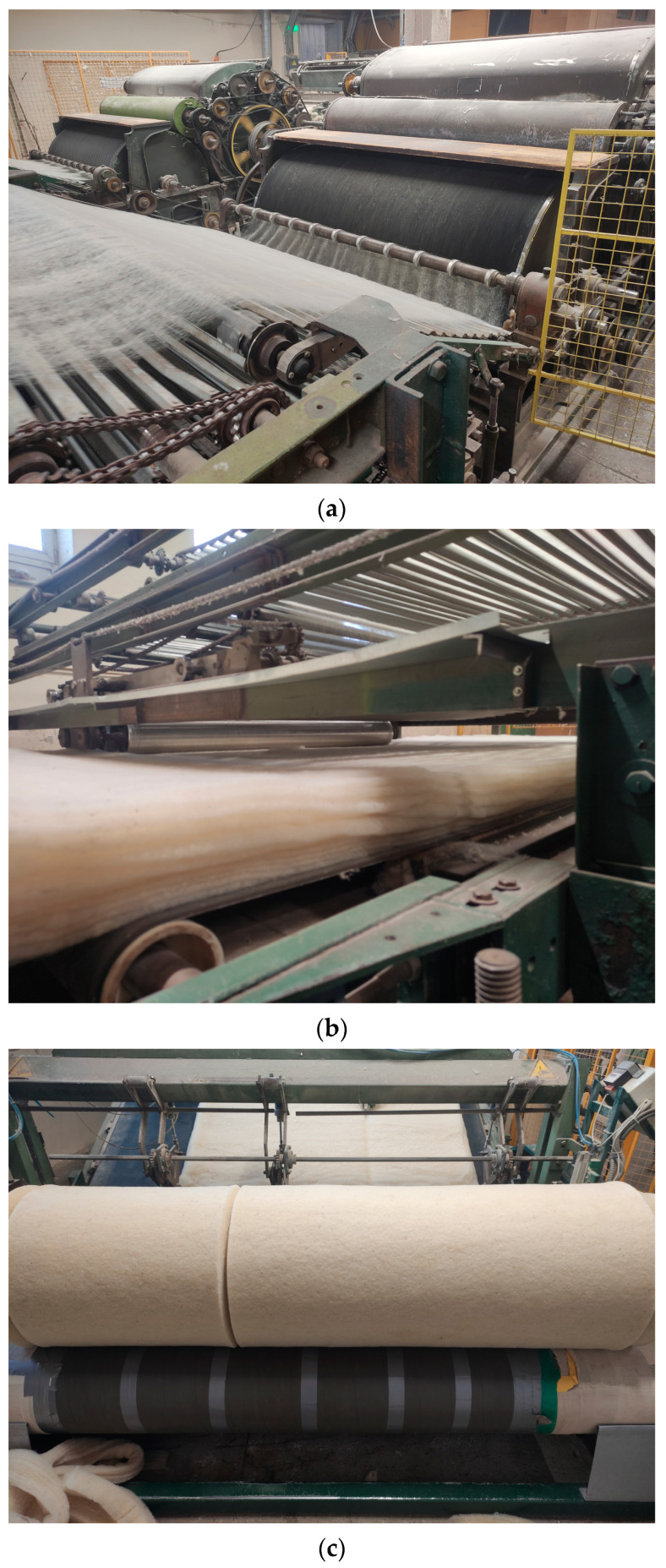
Production stages of soft fibreboards: (**a**) preparation of the carded layer in the carding machine; (**b**) formation of the mat with the folder from carded layers; (**c**) cutting of the prepared mat in the cutting line.

The individual slab layers were produced by a carding machine (Figure 1a), and the resulting layer was laid horizontally with a folder until the required mat thickness was reached (Figure 1b). The resulting mat of the desired thickness was heated in a chamber at 160 °C for 3 min to form contact zones between the wool fibres and the polylactide. The effectiveness of the contact zones between the wool fibre and polylactide under different thermal treatment regimes was evaluated using a scanning electron microscope JEOL JSM-7600F (JEOL, Tokyo, Japan). The thermal treatment regime, i.e., the temperature and the duration of the treatment in the chamber, were chosen so that the polylactide covers the fibres of the wool waste well and binds them together but does not form accumulations of molten polylactide. The mat in the heating chamber was pressed with a rotating shaft to obtain a product of the desired thickness. The mat exiting the heating chamber suddenly cools and the product assumes a stable shape. The cooled mat was cut into boards of 0.5 to 2 m in width and 1 m in length (Figure 1c).

### 2.4. Determination of the Sound Absorption Coefficient

The sound absorption coefficient was established according to ISO 10534-1 [30] using the impedance tube method. With this method, it is possible to readily obtain measurements of normal incidence parameters using small specimens. The Kundt tube had an internal diameter of 85 mm and a length of 1000 mm. Before use, the test equipment was checked by a series of tests. The working frequency range was from 125 to 2000 Hz. Our measurements only covered frequencies up to 2000 Hz, as our tube diameter was 85 mm.

### 2.5. Methods of Processing the Experimental Data

For processing the experimental data and evaluation of their reliability, mathematical-statistical methods, along with the program “STATISTICA” were used [31]. The sound absorption coefficients of the specimens under different frequencies were approximated by Equation (1) when the density of the material was 18 kg/m^3^ and Equation (2) when the density of the material was 40 kg/m^3^:

(1) $$\bar{\alpha }=b_{0}+b_{1}\cdot f,$$

and

(2) $$\bar{\alpha }=b_{0}\cdot \left(1-\exp\left(-b_{1}\cdot f^{b_{2}}\right)\right)$$

where *ᾱ* is the sound absorption coefficient; *ƒ* is the frequency, Hz; *b*_0_, *b*_1_, and *b*_2_ are constant coefficients calculated according to experimental data using the least-squares method [32].

The standard deviation *S_r_* (a measure of the amount of variation or dispersion of a set of experimental values) that we have determined according to Equation (3) [32]:

(3) $$s_{r}=\sqrt{\frac{\sum_{i=1}^{i=n}{\left(Y_{xi}-{\bar{Y}}_{xi}\right)}^{2}}{n-m}}$$

where $Y_{xi}-\left(\alpha \right);{\bar{Y}}_{xi}-\left(\bar{\alpha }\right)$—are the actual and the *i*-value of the resulting characteristic calculated by Equations (1) and (2); n is the number of test results; m is the number of estimated constant parameters.

## 3. Results and Discussion

### 3.1. Analysis of Woollen Yarn Waste

**Figure 2:**
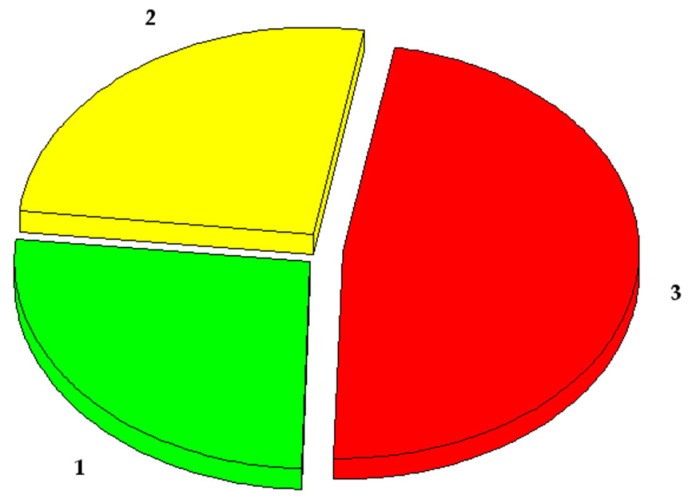
Woollen yarn waste fibres fractions by length: 1—short fibres and dust, fibres length < 10 mm (26.0%); 2—medium length fibres, fibres length 10–30 mm (26.3%); 3—long fibres, fibres length > 30 mm (47.7%).

The length of fibres and their amount in the raw material as well as the composition of non-fibrous matter and different fibre content were determined. In the first case, by combing raw materials, three fractions of fibres were obtained and their masses were determined (Figure 2). In the second case, the amounts of non-fibrous matter and different fibre content were determined (Table 1).

Research has shown that the processing of woollen yarns generates about 74% of the waste that is suitable for the production of acoustic boards. The remaining 26% of the waste is very fine fibres and dust of various origins that is not suitable for the production of boards, but it could be used as fuel in cogeneration plants, as fertiliser in agriculture, etc. A detailed analysis of the long and medium-length fibres shows that it contains about 95% by weight of sheep’s wool fibres and about 5% are fibrous and non-fibrous impurities. About 3.3% of this mass is synthetic yarn and salts formed during the yarn production process, and the remaining 1.7% are the remaining impurities after processing raw sheep’s wool.

### 3.2. Production of Soft Fibrous Boards

**Figure 3:**
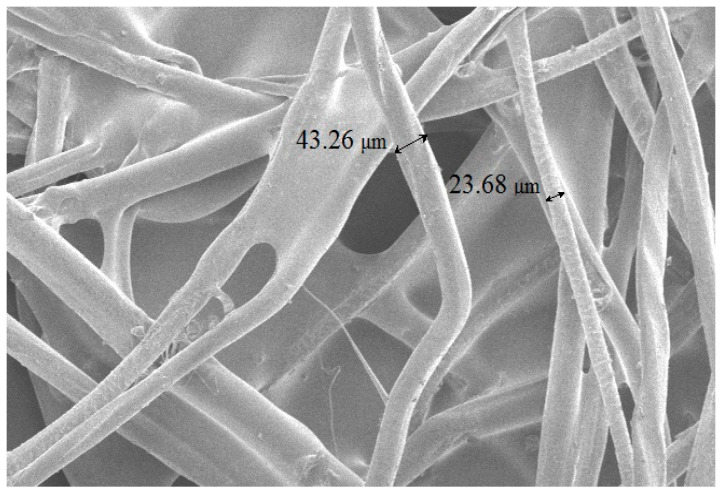
Woollen yarn waste fibres bonded with molten polylactide.

Polylactide was used to join the individual fibres to make soft acoustic boards. When heated to 160 °C, the polylactide melts, and the liquid mass envelopes the fibres during melting. As the mass cools, strong bonds are obtained between the wool fibres and the polylactide (Figure 3).

Four series of products were produced. The description of the products is given in Table 2. According to previous studies [33], 87% of woollen yarn waste and 13% of polylactide were chosen.

### 3.3. Acoustical Characteristics

**Figure 4:**
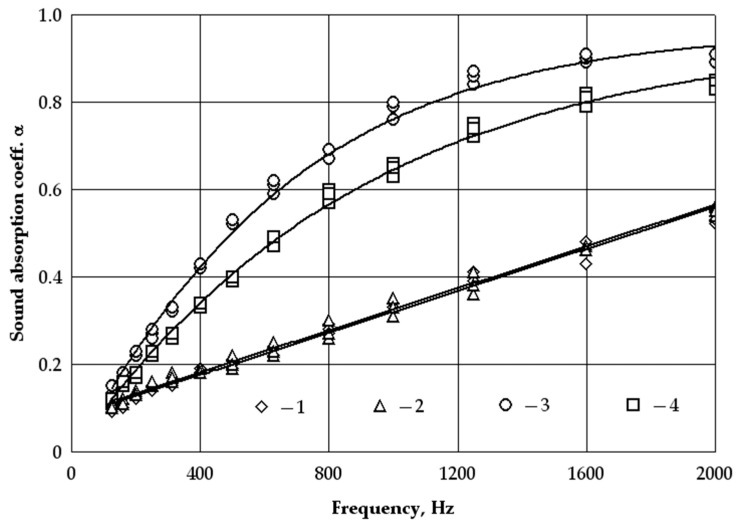
Graphical interpretation of the regression line (Equations (4)–(7)) for the dependence of the sound absorption coefficient on the frequency of the waste product from woollen yarn at different densities (kg/m^3^) and thicknesses (mm): 1—◊ (18 kg/m^3^–40 mm), 2—∆ (18 kg/m^3^–60 mm), 3—○ (40 kg/m^3^–40 mm), 4—□ (40 kg/m^3^–60 mm).

Most fibrous materials are described by a good sound absorption coefficient. In the current work, the sound absorption coefficients of the waste products of woollen yarn were tested with densities of 18 and 40 kg/m^3^ and thicknesses of 40 and 60 mm (Figure 4).

Figure 4 shows that at a product density of 18 kg/m^3^ and thicknesses of 40 and 60 mm, the sound absorption coefficient varies from about 0.11 to 0.56 at sound wave frequencies of 125 to 2000 Hz. At the same product thicknesses and the frequency of the sound waves and a product density of 40 kg/m^3^, the sound absorption coefficient varies from approximately 0.12 to 0.93. Furthermore, a mathematical statistical analysis of the experimental data was performed. It showed that at a density of 18 kg/m^3^ and thicknesses of 40 and 60 mm, the sound absorption coefficient of the woollen yarn waste product increases linearly with increasing frequency from 125 to 2000 Hz. The variation of the sound absorption coefficient over the frequency range of 125 to 2000 Hz can be expressed as a linear dependence (Figure 4, Table 3, Equations (4) and (5) derived from Equation (1)).

Meanwhile, at densities of 40 kg/m^3^ and thicknesses of 40 and 60 mm, there is a sharp increase in the sound absorption coefficient, which lasts up to about 1600 Hz. As the sound frequency continues to increase from 1600 to 2000 Hz, the variation in the sound absorption coefficient remains small. Such a variation in sound absorption coefficient can be described by an exponential equation (see Figure 4, Table 3, Equations (6) and (7) derived from Equation (1)).

**Figure 5:**
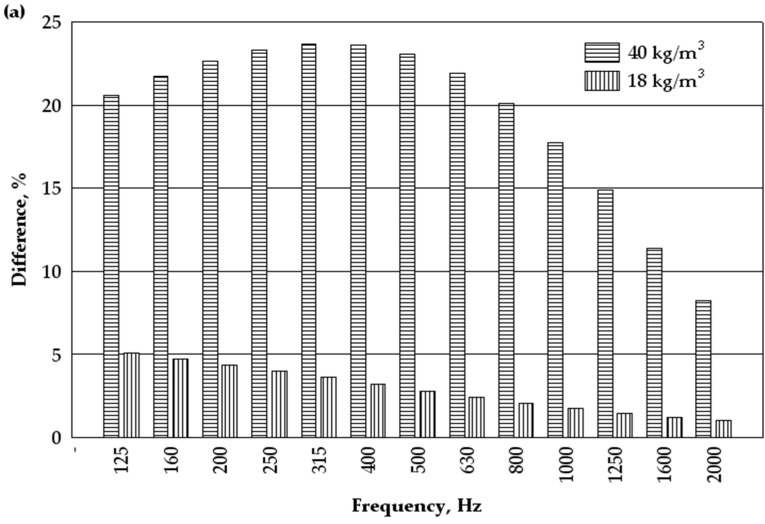

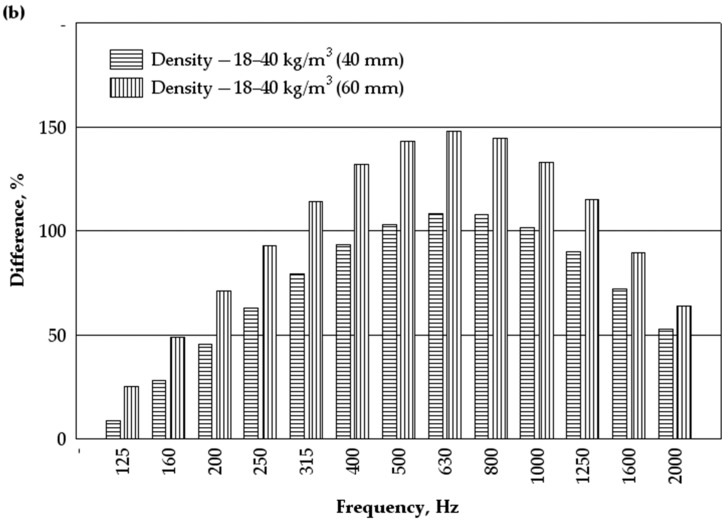
Sound absorption coefficients of the waste products of woollen yarn: (**a**) Difference at different densities (kg/m^3^) and thicknesses (mm)—(18 kg/m^3^ and 40–60 mm) and (40 kg/m^3^ and 40–60 mm); (**b**) Difference at different densities (kg/m^3^) and thicknesses (mm)—(18–40 kg/m^3^ and 40 mm) and (18–40 kg/m^3^ and 60 mm).

For subsequent processing of the results, all data were grouped into two groups according to density and thickness (Figure 5). First, a comparison of the results was made based on the values of the regression equations for specimens of the same density but different thicknesses (Figure 5a). According to the corresponding equations, differences in the values of the sound absorption coefficients were obtained (Equation (4)—18 kg/m^3^–40 mm and Equation (5)—18 kg/m^3^–60 mm; Equation (6)—40 kg/m^3^–40 mm; Equation (7)—40 kg/m^3^–60 mm). Later, according to the corresponding regression equations, a comparison of the results was made for specimens of the same thickness but different densities and differences in sound absorption coefficients were obtained (Equation (4)—18 kg/m^3^÷40 mm and Equation (6)—40 kg/m^3^—40 mm; Equation (5)—18 kg/m^3^ —60 mm and Equation (7)—40 kg/m^3^—60 mm, Figure 5b).

Analysis of the experimental results shows (Figure 5a) that at a product density of 18 kg/m^3^ and thicknesses of 40 and 60 mm, the differences in the sound absorption coefficient at the sound wave frequencies from 125 to 2000 Hz vary from about 5 to 1%. Meanwhile, at a product density of 40 kg/m^3^ and thicknesses of 40 and 60 mm, the sound absorption coefficient at the same frequency difference changes from about 24 to 8%. It can be stated that at a product density of 18 kg/m^3^ for thicknesses of 40 and 60 mm, the difference in the sound absorption coefficient is insignificant. At the same time, the difference in the sound absorption coefficient is significant at a product density of 40 kg/m^3^ for a thickness of 40 and 60 mm.

Comparing the sound absorption coefficients at a thickness of 40 mm (Figure 5b) and the product densities ranging from 18 to 40 kg/m^3^ we can observe differences at all frequencies of the sound wave. We can observe significant differences at frequencies from 500 to 1000 Hz, which makes up more than 100%.

Similarly, a comparison of the sound absorption coefficient at a thickness of 60 mm and densities ranging from 18 to 40 kg/m^3^ shows a significant difference between 315 and 1250 Hz, which also exceeds 100%.

The literature analysis shows that fibrous and porous materials have good acoustic properties. Researchers have determined that natural fibres have very good sound absorption, especially in the mid to high-frequency range [34]. These researchers found that the sound absorption coefficient ranged from 0.10 to 0.94 and 0.15 to 0.94 for sheep wool specimens with densities of 40 kg/m^3^ (40 and 60 mm thick) at frequencies ranging from 125 to 2000 Hz. Other researchers [35] determined the sound absorption coefficient of sheep wool specimens, varying in thickness from 20 to 60 mm, which varied from 0.55 to 0.85 in the frequency range of 100 to 3150 Hz. According to investigations [36,37], wool products have good acoustic properties due to their very small cavities.

Comparing our results with those of other scientists, we can state that the results are very similar. However, it is sometimes difficult to compare the results obtained with those of other scientists due to the different choices of parameters and measurement methods [34,38,39,40]. The sound absorption coefficient of fibrous products also depends on different factors: material density, thickness, structure, pore size, fibre diameter and orientation, airflow resistivity, etc. [34,40,41,42,43].

The sound absorption coefficient of the woollen yarn waste product was determined at frequencies from 125 to 2000 Hz, but in Table 4, the results for only four frequency ranges (250, 500, 1000, and 2000 Hz) are given. These frequency ranges are used to determine the noise reduction coefficient (NRC) [34,40,44], which is applied to compare different materials.

A recalculation of the experimental data showed that at a density of 18 kg/m^3^ and a thickness of 40–60 mm, the NRC is equal to 0.35. Therefore, materials of this density cannot be used as effective sound-absorbing products. However, when the woollen yarn waste product has a density of 40 kg/m^3^ and a thickness of 40–60 mm, the NRC ranged from 0.55 to 0.65. Materials with this density could be classified as sound-absorbing.

The values obtained were compared with the results obtained by other authors. Researchers [35,41] found that the noise reduction coefficients of specimens of wool fibres having a density of 40 kg/m^3^ and thicknesses of (40–60 mm), were in the range of 0.55–0.70, and our values for this factor at 40 kg/m^3^ and thickness (40–60 mm) were 0.55–0.65. A comparison of the experimental data showed that the difference at the 60 mm product thickness reaches 7.7%.

The increase or decrease in the values of the sound absorption coefficient may be determined by different technological parameters of the processing of the fibres or the initial raw material.

## 4. Conclusions

About 74% of the waste from the production of woollen yarn according to the dimensional parameters could be used as ready raw material for the production of soft acoustical mats or boards.

The acoustic performance of soft boards made from woollen yarn waste is very similar to that of classic boards or sound insulation products made from renewable resources. Woollen yarn waste products of 18 kg/m^3^ density were found to be characterized by poor sound absorption in all of the analysed sound frequency ranges. The sound absorption coefficients of such products ranged from 0.1 to 0.6. Meanwhile, by increasing the density of the material by almost twice, that is, to 40 kg/m^3^, significant growth of the sound absorption coefficient over the entire sound frequency range was observed. In this density range, the value of the sound absorption coefficient ranged from 0.4 to 0.9. The noise reduction coefficient shows that poor sound-absorbing properties are observed at the material density of 18 kg/m^3^, and the coefficient reaches a value of just 0.35. At a density of 40 kg/m^3^, sound absorbing properties are good, and the noise reduction coefficient reaches a value of 0.65.

Further studies on the evaluation and optimisation of building material properties such as settlement, tensile strength, and durability, as well as the fire resistance of the material, will be required before sound-absorbing mats could be used in buildings and construction.

## Figures and Tables

**Table 1:** Description of woollen yarn waste.

Quantity of Fibrous and Non-Fibrous Matter in Waste, Mass %				
Wool Fibres	Organic Fibres	Other Fibres	Salts, etc.	Fats
95.0	0.6	0.5	2.8	1.1

**Table 2:** Description of the prepared specimens.

Test Specimens Indicators		Composition of the Specimens	
Thickness of the Specimen, mm	Density of the Specimen, kg/m^3^	Woollen Waste, mass %	Polylactide, mass %
40	18	87	13
60	18		
40	40		
60	40		

**Table 3:** Statistical processing results of sound absorption of waste product from woollen yarn.

Number of Regression Equations	Number of Tests (Estimations)	Values of Constant Coefficients of Equations (4)–(7)			${}_{R_{\alpha \cdot f}^{2}}^{}$	*S_r_*
		$b_{0}$	$b_{1}$	$b_{2}$		
(4)	39	0.08618	0.00024		0.9955	0.01315
(5)	39	0.08054	0.00024		0.9879	0.01543
(6)	39	0.953021	0.000971	1.022208	0.9971	0.01396
(7)	39	0.963258	0.00087	1.084321	0.9949	0.02022

**Table 4:** Noise reduction coefficients of different product compositions.

Materials Density, kg/m^3^	Thickness, mm	Frequency of Sound Waves, Hz				NRC
		250	500	1000	2000	
		Sound Absorption Coefficient				
18	40	0.15	0.20	0.33	0.55	0.35
18	60	0.15	0.20	0.33	0.53	0.35
40	40	0.22	0.40	0.65	0.84	0.55
40	60	0.27	0.52	0.78	0.89	0.65

## Data Availability

Not applicable.
